# Supplementary material for: HuBMAP Data Portal: a resource for multimodal spatial and single-cell data of healthy human tissues
Source: Nat Commun. 2026 Nov 2;17:11125. doi: 10.1038/s41467-026-77835-z (PMC13728199; doi:10.1038/s41467-026-77835-z)
Supplement: Supplementary file 2 [file 41467_2026_77835_MOESM2_ESM.pdf]

nature portfolio

Peer Review File

HuBMAP Data Portal: a resource for multimodal spatial and single-cell data of healthy human tissues

Corresponding Author: Dr Morgan Turner

This file contains all reviewer reports in order by version, followed by all author rebuttals in order by version.

Version 0:

Reviewer comments:

Reviewer #1

(Remarks to the Author)
Please see uploaded reviewer comment file, which contains examined portal images as examples.

(Remarks on code availability)
Please see uploaded reviewer comment file.

Reviewer #2

(Remarks to the Author)
I co-reviewed this manuscript with one of the reviewers who provided the listed reports. This is part of the Nature Communications initiative to facilitate training in peer review and to provide appropriate recognition for Early Career Researchers who co-review manuscripts.

(Remarks on code availability)
They provide detailed and organized code. And they also provide R and jupyter notebook script example for users to practices.

Reviewer #3

(Remarks to the Author)
This manuscript describes the development and current state of the HuBMAP Data Portal as a repository for multi-modal spatial and single-cell data from healthy human tissues. The authors detail the infrastructure, including uniform processing pipelines, visualization, and cloud-based workspace environments. The scale of the engineering effort is impressive, hosting over 5000 datasets and providing a valuable centralization of complex spatial omics data. The manuscript is well-structured and documents a resource that will likely be important for the spatial biology community.

Overall, I find the resource description strong and the infrastructure robust, but there are significant issues regarding the framing of the data's representativeness and the clarity of its presentation that should be addressed before publication. My main concerns are as follows.

1. The manuscript emphasizes the utility of 'uniform processing pipelines' for harmonizing data across the portal. However, the distinction of processing between bulk, single-cell transcriptomics, and spatial proteomics is not made sufficiently clear, leading to potential confusion regarding the analysis provided. The text notes that cell type annotations were generated using Azimuth, but explicitly links this only to 'RNA-seq and Slide-seq' datasets. This implies that the vast majority of spatial datasets (e.g., CODEX, PhenoCycler and Visium) lack uniform cell type annotation. Tools like Azimuth are designed for transcriptomic and non-imaging data annotation. If the authors intended to imply that 'uniform processing' extends to spatial proteomics, they must clarify what specific methods were used, as transcriptomic tools are not directly applicable to imaging data. If a uniform strategy was indeed attempted for spatial data, there is a high risk of suboptimal annotation. Even between single-cell and bulk data, the “uniform pipeline” is different. For bulk data for example, cell-type deconvolution is a commonly used tool but it is certainly not needed for single-cell. Please explicitly state in the Methods or Results whether and what methods bulk, single-cell and spatial data undergo, respectively. Please qualify the term 'uniform processing' to avoid the

misleading impression that spatial datasets possess the same level of annotation as the single-cell RNA datasets.

2. The labeling of data types in Figure 2h and Figure 3 is imprecise and potentially confusing for a technical audience. The authors use the label RNAseq to describe a category that appears to largely consist of bulk, single-cell and single-nucleus RNA sequencing data. Normally, RNA-seq implies bulk sequencing. Furthermore, the distinction between multimodal assays and their constituent parts is ambiguous in the bar plots. It is unclear whether the ATACseq bar includes the chromatin accessibility component of multimodal assays like SNARE-seq2 or 10x Multiome, or if these are counted separately. The figures should be relabeled to explicitly distinguish between bulk and single-cell modalities, and the caption should clarify how multimodal datasets are quantified to avoid perceived double-counting.

3. The manuscript describes the repository as a spatial reference atlas, but it lacks critical descriptive metadata regarding the nature of the spatial samples. For example, the text does not distinguish between Whole Slide Images (WSI), Biopsies, and Tissue Microarrays (TMAs). A 'spatial atlas' constructed primarily from TMAs (which lack anatomical gradients and macro-structure) has very different utility compared to one built from whole-tissue sections. Please clarify in the text and metadata figures (e.g., Figure 2) the proportion of spatial datasets that are TMAs vs. whole sections. Similarly, for each dataset, the authors should also clarify how many slices there are. Overall, more detailed statistics for spatial data is needed.

4. The manuscript lacks a sufficient discussion of the limitations imposed by the data skewness such as demographic (a big amount of samples are labeled as “unknown” and overwhelmingly, most samples are “white”). While the Discussion section touches on spatial sparsity, it glosses over the biological implications of the demographic imbalances. For a resource intended to serve as a healthy reference for disease studies or a training set for computational models, the risks of using such a demographically narrow cohort as a control group are significant. I strongly suggest adding a limitation subsection that explicitly addresses these gaps and warns users about the potential artifacts arising from the lack of diversity and uneven organ sampling.

Overall, the HuBMAP Data Portal is a commendable technical achievement that advances the accessibility of spatial omics data. However, for the manuscript to serve as an accurate description of the resource, the authors must address the overstatements regarding the atlas's comprehensiveness and improve the precision of their data visualization labels. With these clarifications, the paper would be a valuable contribution to the field.

(Remarks on code availability)

Reviewer #4

(Remarks to the Author)
In the manuscript tittled ‘ HuBMAP Data Portal: A Resource for Multi-Modal Spatial and Single-Cell Data of Healthy Human Tissues’ the authors present their biological data portal/repository containing data generated in the HuBMAP (NIH, US) program. Data bases and data searches (incl HuBMAP) have been extensively evaluated by the NIH and this reviewer mainly evaluates the manuscript and the provided information, the novelty and impact and position within the landscape of life science data resources. This reviewer is not a member of the HuBMAP consortium and has not used the presented data portal prior to this evaluation task and only evaluated the examples provided by the authors.

Impact and gap: Major challenges in data driven life science are findability, standardization of data acquisition and (primary) analysis, quality control and availability of meta data on the origin of the used samples. There are several data repositories (GEO database, Array express) and data portals available including (public, non-profit) efforts that can search across multiple repositories like OmicsDI (EU). It is important that novel initiatives are not ‘yet another data resource’ but provide additional benefits and improvements to facilitate high quality life science research now and in the future. The collected data is impressive (methods, datasets) but is restricted to data generated within the HuBMAP consortium which has the advantage of standardized methods, sample description QC etc. The disadvantage of this restriction are the limited possibilities to apply HuBMAP data analysis and data visualization tools combining data from various resources (though implemented tools are opensource).

The manuscript is well written, but sometimes the detailed information is less relevant for readers not member of the HuBMAP consortium and there are several topics that would benefit from more information. Some of these are listed below. This manuscript/report could benefit from some use case examples.

1) HuBMAP data on the portal: provides 22 different data types of in total 5000+ samples.

- How can a user search for target genes in antibody and/or probe-based assays based on gene or protein panels? Could the authors describe how to get perform a gene-centric search finding datasets that contain information on specific genes or gene products.
- Why do the authors include race in donor metadata as in biology human races do not exists? Ethnicity would be a more acceptable term.

2) Data-types:
- It is difficult to find the rationality behind the choice for samples, number of samples and what methods applied to selection of tissue samples. Even though this is a large collection of samples; some tissues are overrepresented (780 right lung samples) where other tissues, like liver and brain, have <20 samples. This affects the completeness of the resource and the

applications/ in silico experiments that will be biased by available data. Could the authors present an example analysis types of data mining/analysis that could be performed with the HuBMAP resource.
- Why are CODEX and Phenocycler data annotated differently?

3) Data Submission:
- Since only members of the HuBMAP consortium can upload data it is not that interesting for the reader/user not part to consortium to read the details about data submission. I link to the protocols would be sufficient. It would also be a great value for the reader/user to learn if some HuBMAP procedures are adapted from already used standards for data sharing.
4) Data Ingestion:
- This step includes validation, reorganization, processing, quality assurance and quality control. The authors should also explain their procedures for implementing new standards in processing and quality control. In the Provenance paragraph the authors describe that data can is versioned if data will be processed using new pipelines. It would be of great help for the reader to understand how this will be applied. Will all data be reprocessed at the same time to ensure the compatibility?

5) HuBMAP data organization:
- Multi-omics data integration is extremely challenging and even integration of similar data like single cell or single nuclei transcriptomics data from different experiments is challenging. It is appreciated that HuBMAP is providing data integration and data analysis pipelines. The authors should provide an example on how their pipeline could and should be used and how the results should be interpret and benchmarked for accuracy.

6) User-drive portal design and tool functionality
- It is clear from the efforts that HuBMAP aims to serve the life science community. It would be extremely valuable if the analysis HuBMAP data that results in peer reviewed scientific publications could be reproduced within HuBMAP and the used pipelines would be available. What scientific publications by HuBMAP consortia members involving data in the portal has been processed using the available tools and pipelines. A list us these publications and pipelines would be extremely valuable for the community to get familiar with the benefits and options of the data portal.

(Remarks on code availability)
I have looked a parts of the documentation and code, but it was too much to investigate everything in full detail.

It is a huge collection of scripts and code with sometimes strange names that are a bit difficult to navigate. A worked out example of creating and running a pipeline to answer a biological question would help the user to navigate through the options.

Version 1:

Reviewer comments:

Reviewer #1

(Remarks to the Author)
The authors have satisfactorily addressed my concerns. The revised manuscript is significantly improved and more clear.

(Remarks on code availability)
Due to the huge amount of code in the portal, we were not able to carefully review all the code. But we asked specific questions related to code-generated pictures and segmentation which were satisfactorily addressed.

Reviewer #2

(Remarks to the Author)
I co-reviewed this manuscript with one of the reviewers who provided the listed reports. This is part of the Nature Communications initiative to facilitate training in peer review and to provide appropriate recognition for Early Career Researchers who co-review manuscripts.

(Remarks on code availability)

Reviewer #3

(Remarks to the Author)
Overall, the authors answered my concerns nicely. However, I do have a new concern when reading the response for reviewer 1.

In their response, the authors state only that cell nuclei boundaries are 'segmented using the DAPI channel.' This describes the nuclear input to segmentation rather than the segmentation method itself. Because DAPI is a nuclear stain, segmentation from this channel alone yields nuclear masks rather than whole-cell boundaries; accurate whole-cell segmentation typically requires an additional membrane or cytoplasmic channel (e.g., E-cadherin) to delineate cell borders. Notably, modern segmentation tools such as Cellpose and Mesmer/DeepCell are explicitly multi-channel and do not rely on DAPI alone. If the pipeline performs only DAPI-based nuclear segmentation, this represents a meaningful limitation for a spatial reference

atlas, as restricting boundaries to the nucleus systematically mis-assigns membrane- and cytoplasm-localized marker signal and may compromise per-cell quantification, particularly in densely packed tissues. Please specify the segmentation algorithm used, the input channels it relies on, and whether the outputs are nuclear-only or whole-cell segmentations. If uniform segmentation is genuinely DAPI/nuclear-only, this limitation and its downstream impact on per-cell feature extraction should be explicitly stated in the Methods.

(Remarks on code availability)

Reviewer #4

(Remarks to the Author)
In the revised version of the manuscript 'HuBMAP Data Portal: A Resource for Multimodal Spatial and Single-Cell Data of Healthy Human Tissues' the authors addressed all concerns raised by the reviewers. In my opinion the overall achievement, usefulness and impact on research are great and of great value. These types or large efforts need time to settle and mature, and the current version is not perfect but an has utility and is an excellent foundation to build on.
- I would like to apologize for requesting some information that was already available on the portal. The amount of information was a bit overwhelming, and I would like to thank the authors for their explanation and guidance.
- The use case table (table 1) is very helpful and inspiring on how to use the available data and tools in different life science research settings.
- Data submission paragraph, this now has all the information that a non-HubMAP user needs to understand the data submission process and assess the level and quality of the data.
With the development of AI and AI tools the landscape of life science is changing. This manuscript is a report on a development that started in 2018. The way researchers interact with data and the increasing use of AI agents has changed the data resource landscape and will chance the needs of users. It will be interesting to see the future development and directions of the HuBMAP project and data portal.

(Remarks on code availability)
I have looked at the scripts in the repository, but as also indicated by the authors in their response this is not organized and optimized for sharing so I did not go into the details.

Open Access This Peer Review File is licensed under a Creative Commons Attribution 4.0 International License, which permits use, sharing, adaptation, distribution and reproduction in any medium or format, as long as you give appropriate credit to the original author(s) and the source, provide a link to the Creative Commons license, and indicate if changes were made.
In cases where reviewers are anonymous, credit should be given to 'Anonymous Referee' and the source.
The images or other third party material in this Peer Review File are included in the article's Creative Commons license, unless indicated otherwise in a credit line to the material. If material is not included in the article's Creative Commons license and your intended use is not permitted by statutory regulation or exceeds the permitted use, you will need to obtain permission directly from the copyright holder.
To view a copy of this license, visit https://creativecommons.org/licenses/by/4.0/

Reviewer Responses for *HuBMAP Data Portal: A Resource for Multi-Modal Spatial and Single-Cell Data of Healthy Human Tissues*

REVIEWER COMMENTS

Point-by-point responses to reviewer comments appear in red throughout.

The authors note that dataset and sample counts reported in the manuscript reflect the portal as of June 2026. As HuBMAP enters the final phase of the program, data ingestion is ongoing and counts may increase substantially in the coming months. If permitted, we will update these figures at the proofing stage to ensure the published version reflects the most current data available.

Reviewer #1

Reviewer #1 (Remarks to the Author): Please see uploaded reviewer comment file, which contains examined portal images as examples.
Reviewer #1 (Remarks on code availability): Please see uploaded reviewer comment file.
Reviewer #1 comments copied from the uploaded file, below.

Overall, the manuscript is well-prepared and clearly describes a valuable resource for the research community. The NIH Human BioMolecular Atlas Program (HuBMAP) Data Portal provides an integrated platform for accessing, analyzing, and visualizing multi-modal spatial and single-cell data from healthy human tissues. The authors provide user-friendly handbook and examples for researchers to navigate the platform. Overall, the paper represents an important contribution to advancing multi-scale spatial analysis and data accessibility.

1. The spatial dataset includes cell segmentation data which could be challenging for some tissues. Upon randomly picking and reviewing some data images on the portal, one can easily find inaccurate segmentations which may lead to misleading interpretations, e.g., HBM457.JZFF.434 lymph node CODEX, and HBM238.ZKPC.934 skin Cell Dive shown below. The authors should check every data image to ensure all segmentations are correct.

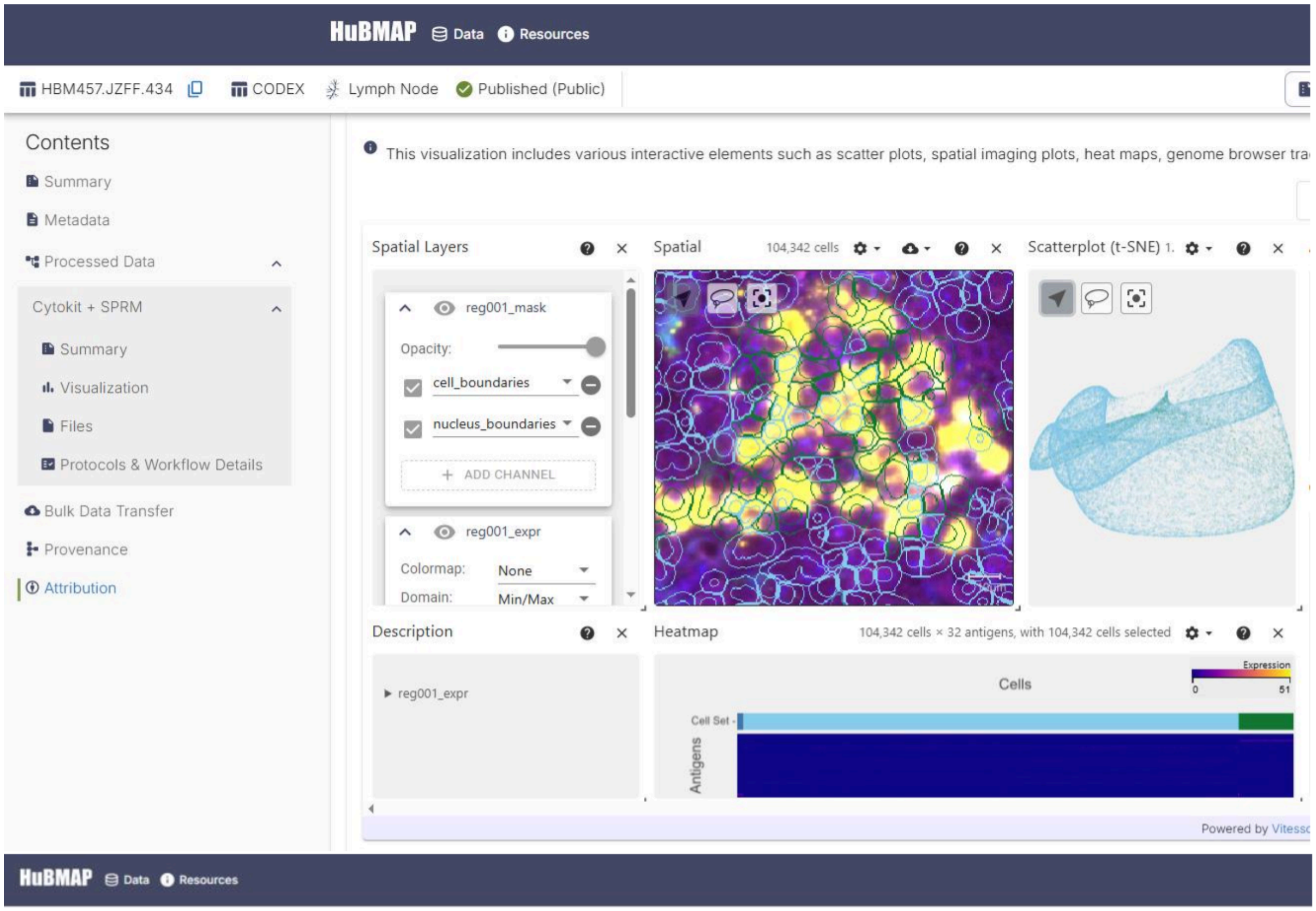

nFeature_RNA

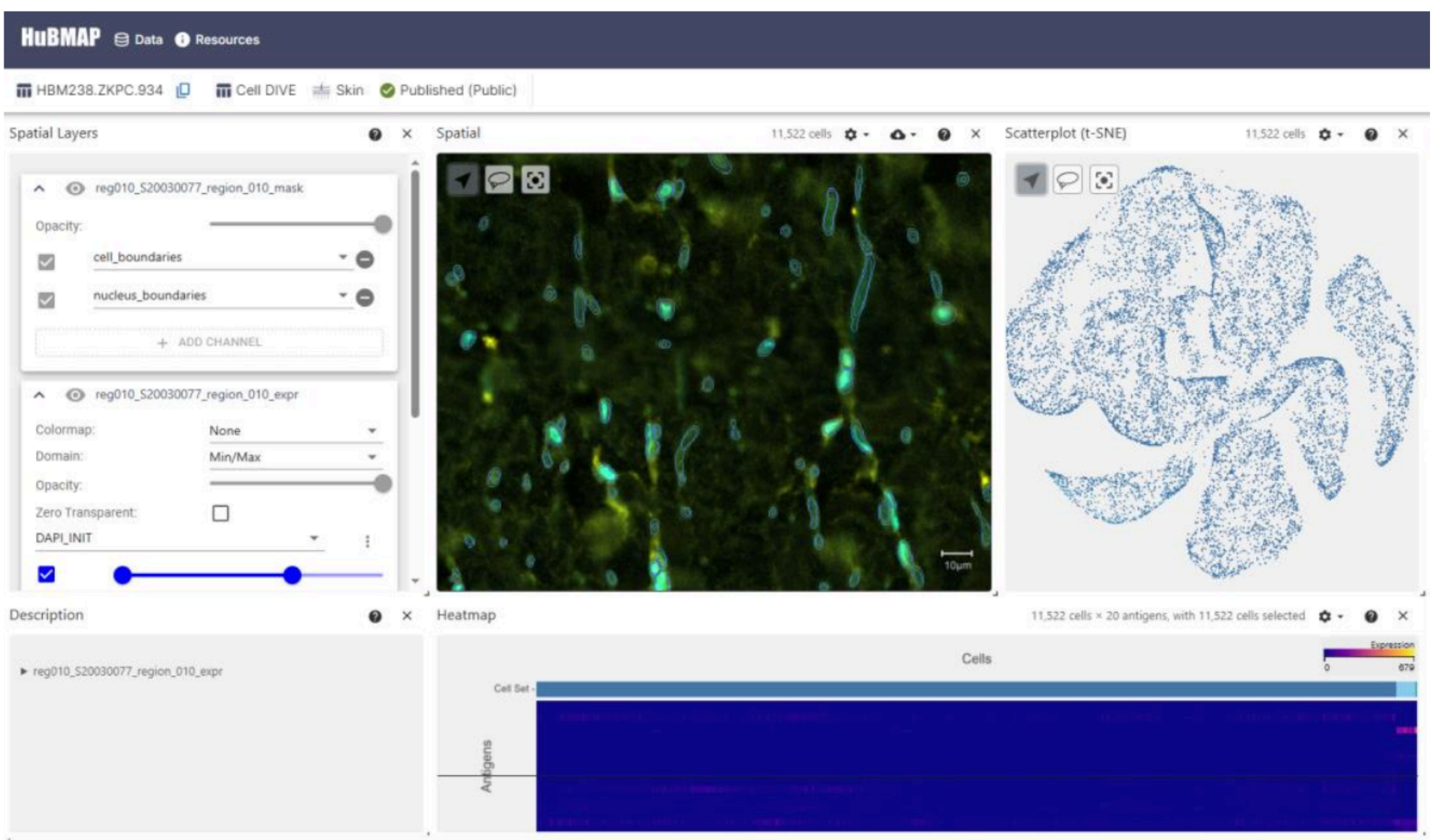

Changes made.

We 1) reviewed & responded to the specific segmentation concerns raised in this comment, 2) made updates to the HuBMAP Data Portal, and 3) revised the manuscript. Details below.

1) Addressing specific segmentation concerns raised:

- For CODEX datasets, cell nuclei boundaries are segmented using the DAPI channel. In the reviewer-provided region of HBM457.JZFF.434 (image below, left), the incorrect channels appear to have been selected. Isolating the appropriate DAPI channel confirms the nuclei segmentations appear correct (right).

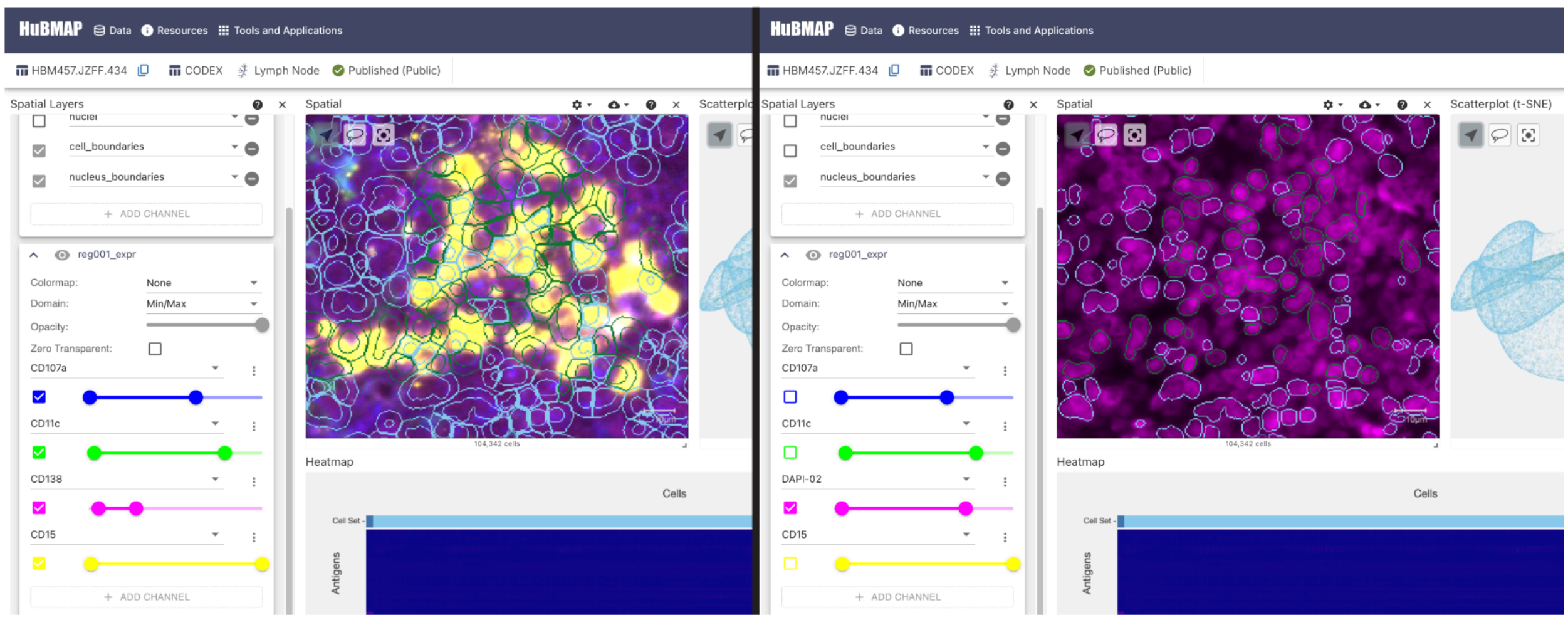

- For Cell Dive datasets, cell nuclei boundaries are segmented using the DAPI image channel. Using the reviewer-provided region of HBM238.ZKPC.934 (image below, left), we confirmed nuclei segmentations appear correct. Isolating the appropriate DAPI channel shows the alignment more clearly (image, right).

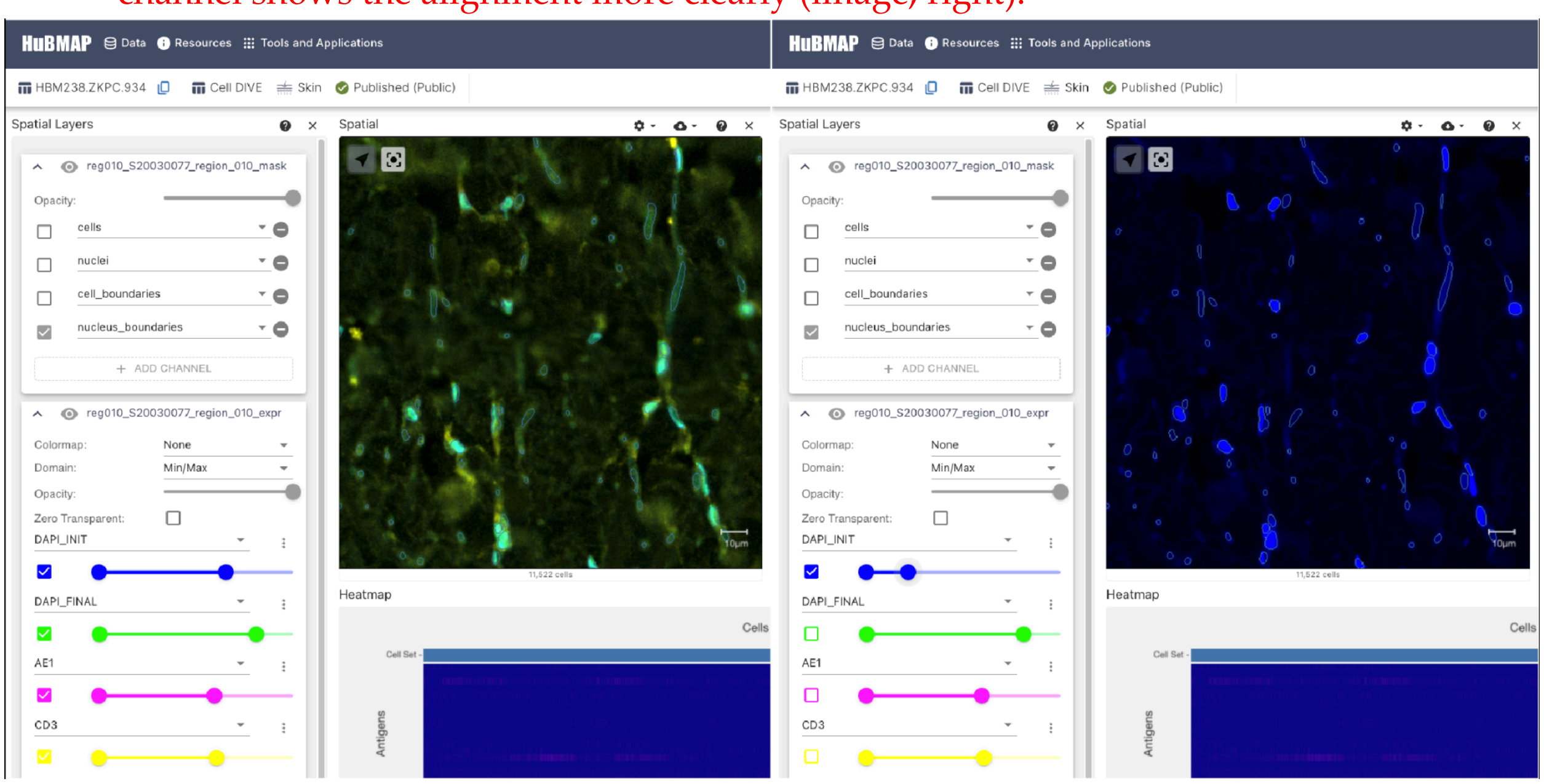

2) We have updated the HuBMAP Data Portal to more clearly surface segmentation information on dataset pages. Segmentation metadata now appears directly below dataset visualizations under the header **Segmentation Channels & Quality** (first image below, red arrow), helping users quickly identify relevant image channels for evaluating segmentation masks. Quality scores from image processing pipelines are also displayed in this location, providing a quantitative indicator of segmentation quality. Additionally, the **Protocols & Workflow Details** section on dataset pages were updated to include assay pipeline descriptions covering details on segmentation workflows, methods, selection criteria, and relevant citations or other resources (second image below).

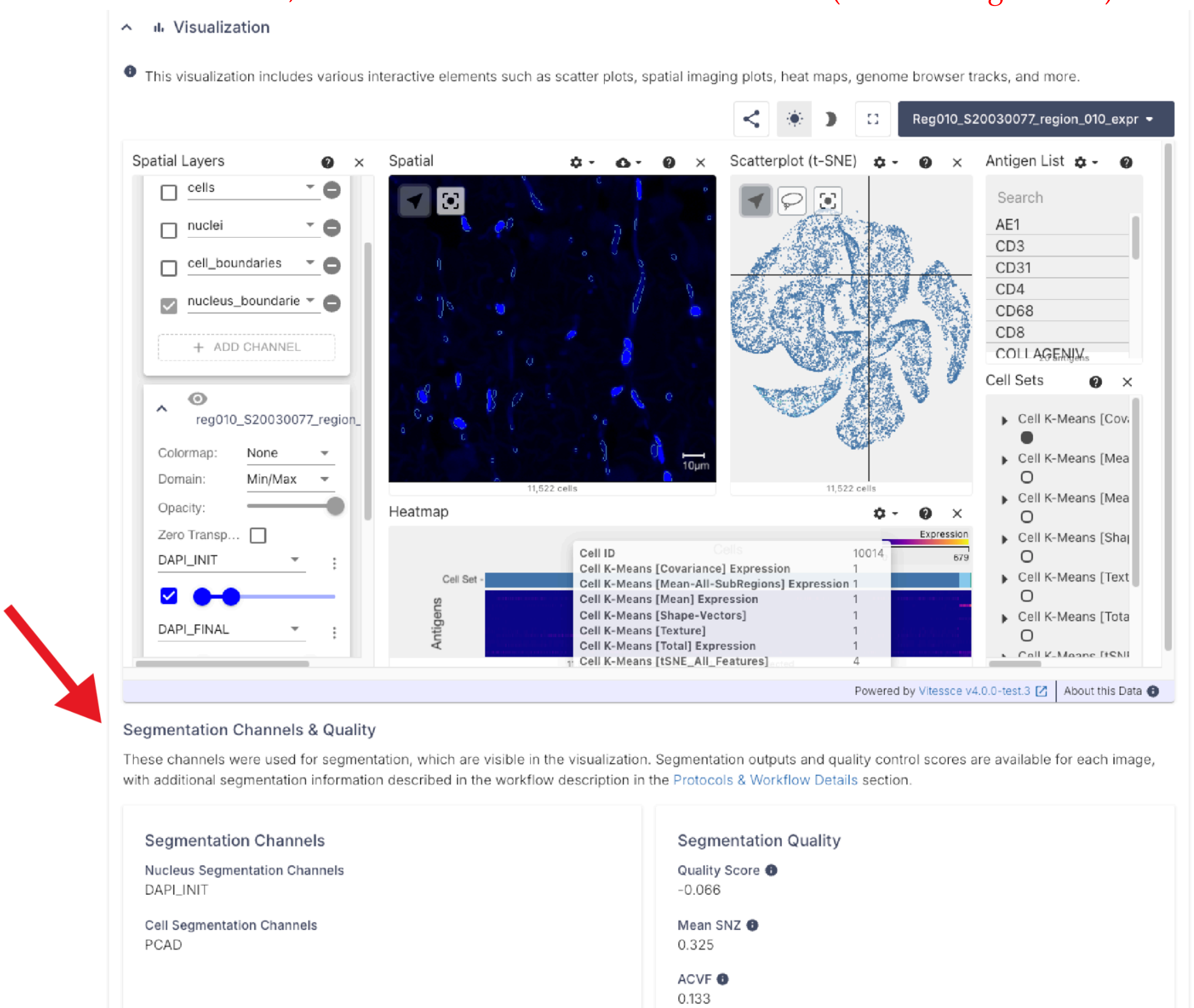

Protocols & Workflow Details

Workflow (v0.0.1)

The CellDive pipeline performs segments nuclei and cells using DeepCell and performs spatial analysis of expression data using SPRM, which computes various measures of analyte intensity per cell, performs clustering based on expression and other data, and computes markers for each cluster. DeepCell was chosen for segmentation based on its performance in an evaluation of various methods across multiple tissues using quality metrics that can be calculated without the need for a human-segmented image for comparison. More information about DeepCell and the associated Mesmer algorithm can be found in the associated publication here. The segmentation masks and expression images are then used for cell type assignment using RIBCA, Stellar, and DeepCellTypes. SPRM is then used for spatial analysis of expression data, including computation of various measures of analyte intensity per cell, clustering based on expression and other data, marker computation per cluster, subclustering of cell types assigned by multiple methods and more.

Collapse Steps (6)

These are the steps executed in the workflow to produce the processed dataset.

Step	Tool	Origin Link	Git Commit	Documentation Link	
1	ingest-pipeline.git (v3.9.2)	https://github.com/hubmapconsortium/ingest-pipeline.git	f534942f	-	-

3) The main text (primarily Results section 1) has been revised to clarify the process by which segmentation methods are chosen, and for what datasets they are available. We have also revised the main text to include detail on the data curation & QA process. However, it is important to note that this paper is not about the underlying processed data.

2. The visualization of the spatial dataset needs to improve. Spatial gene or antigen expression scale overlaps with the section picture, making users hard to see the expression pattern across the section. See example below:

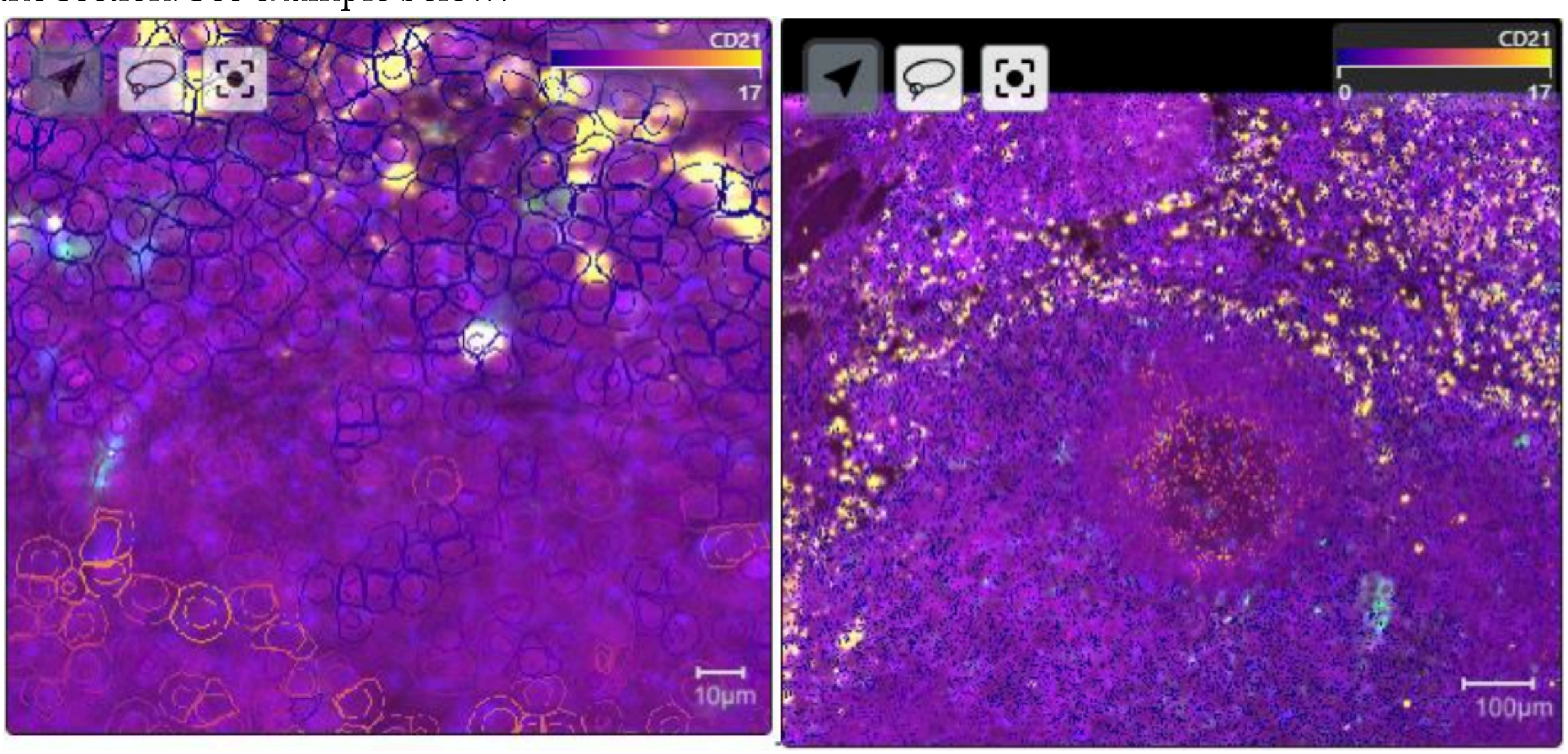

Change made.

The expression scale bar has been removed from the spatial view and no longer overlaps with the underlying image.

In terms of color encoding of the scale, users can freely change color mappings (including to

greyscale) for image channels and gene expressions for better contrast. These options are available on the lefthand spatial layer control menu and in the settings dropdown menu just above the spatial view. The image below demonstrates adjustments to those settings to enhance contrast.

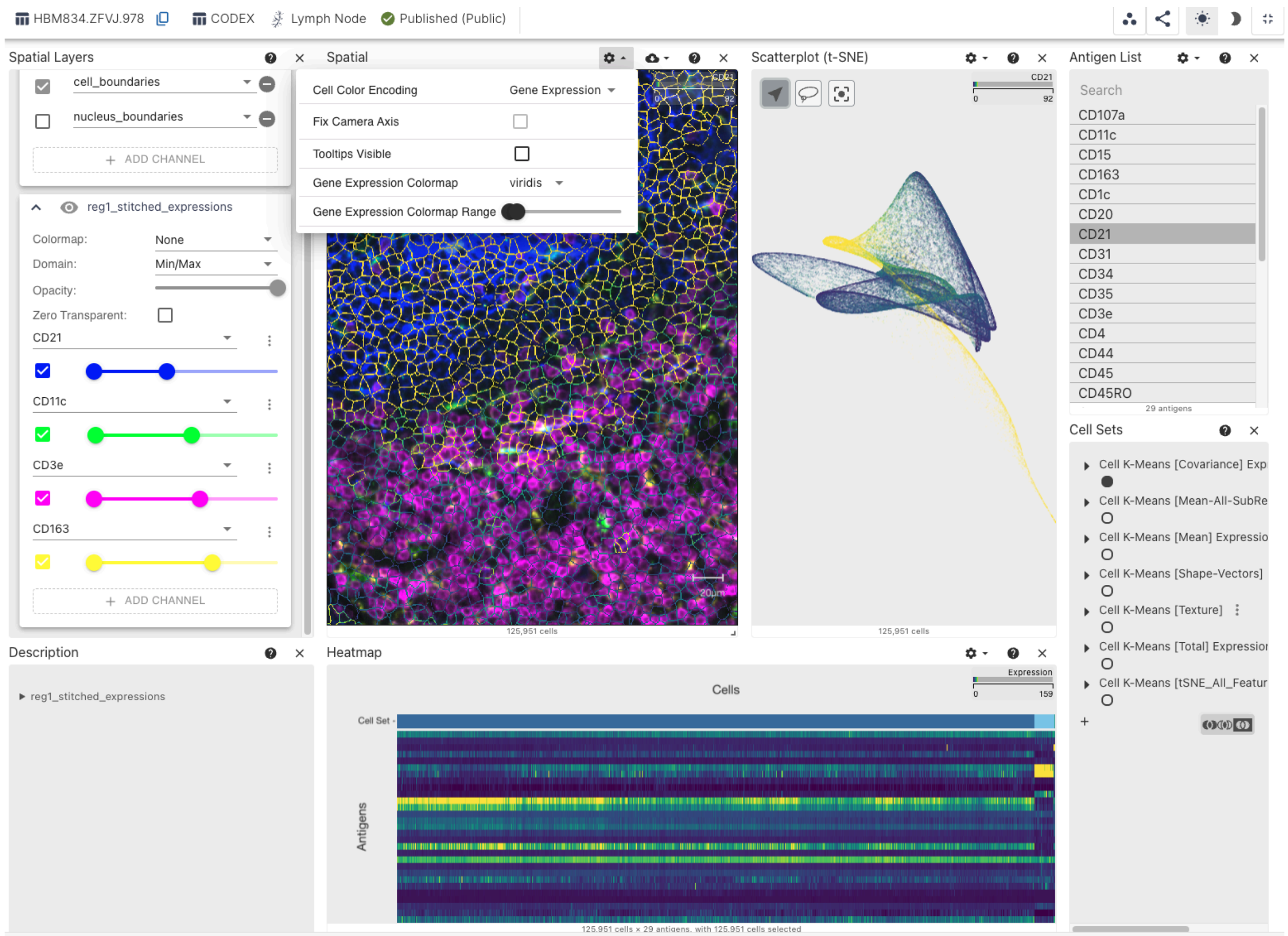

3. Some sample quality is not consistent or needs more description for some datasets. For example, HBM937.RTLZ.357 Uterus Visium data is missing most of the cells compared to the other Uterus Visium dataset. The pictures below are the HBM937.RTLZ.357 and a normal one:

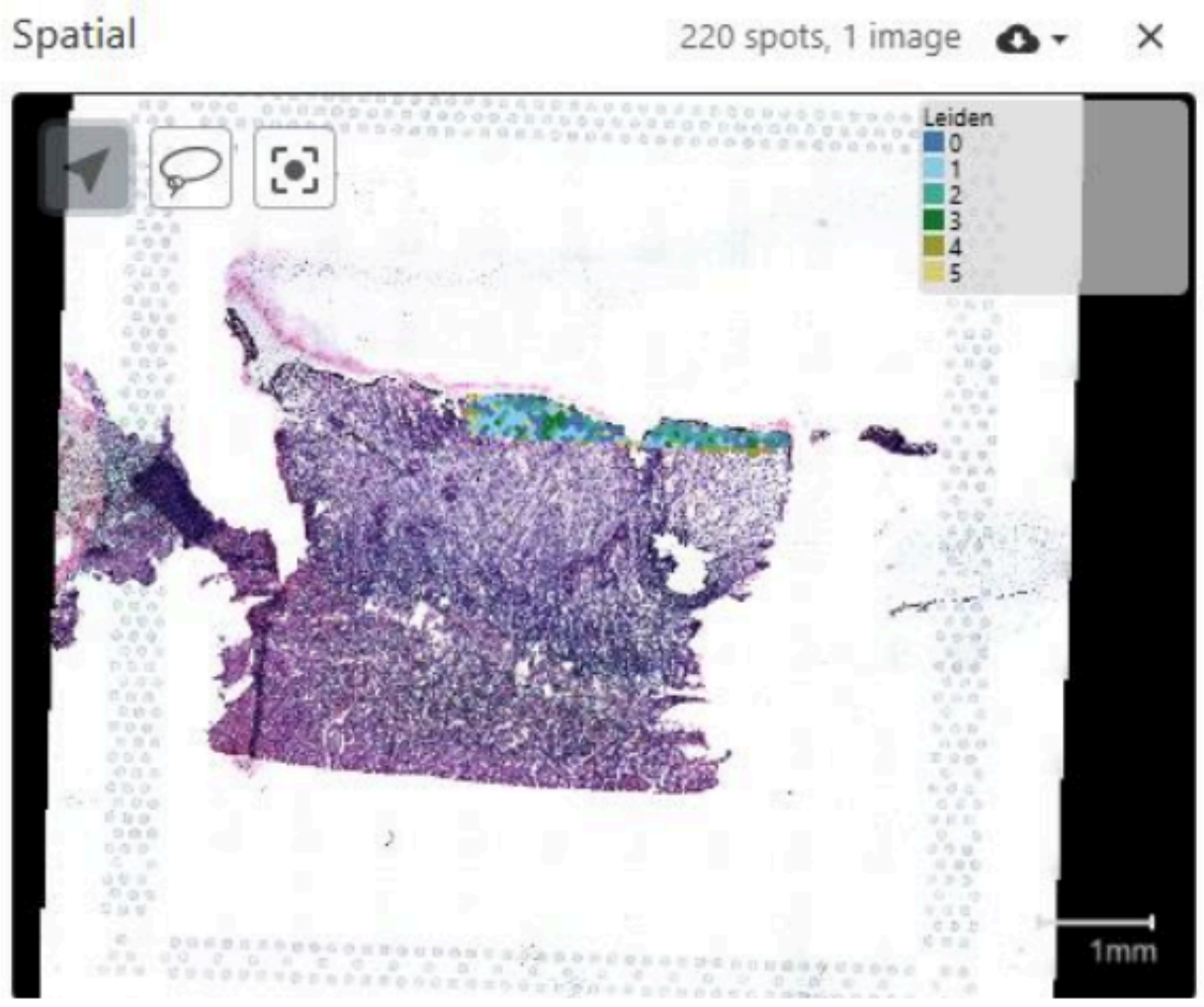

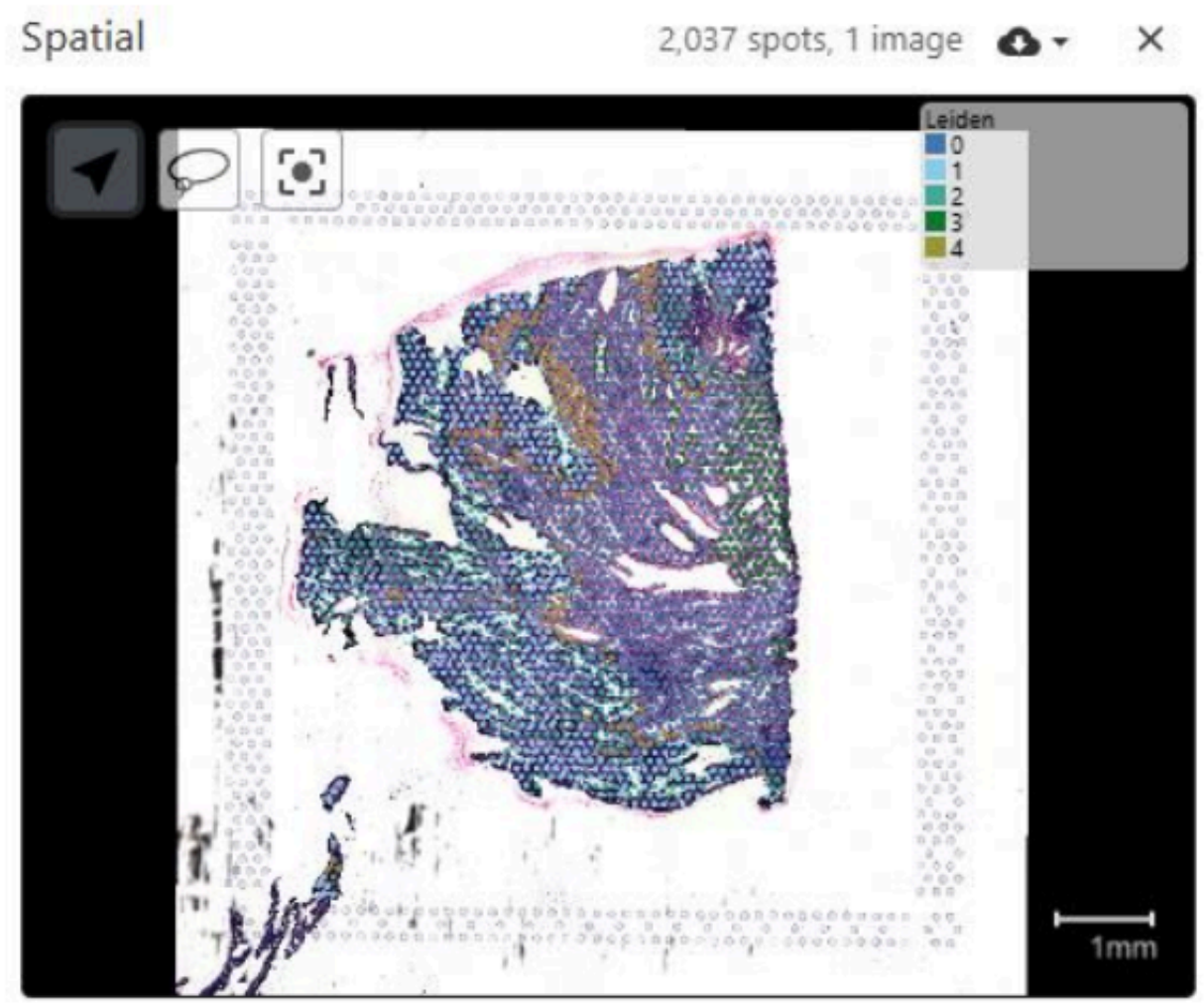

Changes made.

We 1) reviewed & responded to the specific sample quality concerns raised in this comment, and 2) revised the manuscript. Details below.

1) Addressing specific quality concerns raised:
   We thank the reviewer for raising this concern, which we used as an opportunity for review of data errors. The specific dataset HBM937.RTLZ.357 has been identified as having an issue with alignment transformations in the processing pipeline. This dataset has been reprocessed and passed back through QA/QC with review and approval from the original data submitter. The new processed dataset HBM369.KVCG.634 can be seen in the image below, or directly on the portal: https://portal.hubmapconsortium.org/browse/dataset/5278bd6c5df1509737e637a44f92d221?redirected=True&redirectedFromId=HBM369.KVCG.634&redirectedFromPipeline=Salmon+%2B+Scanpy#section-salmon%2bscanpy-published.

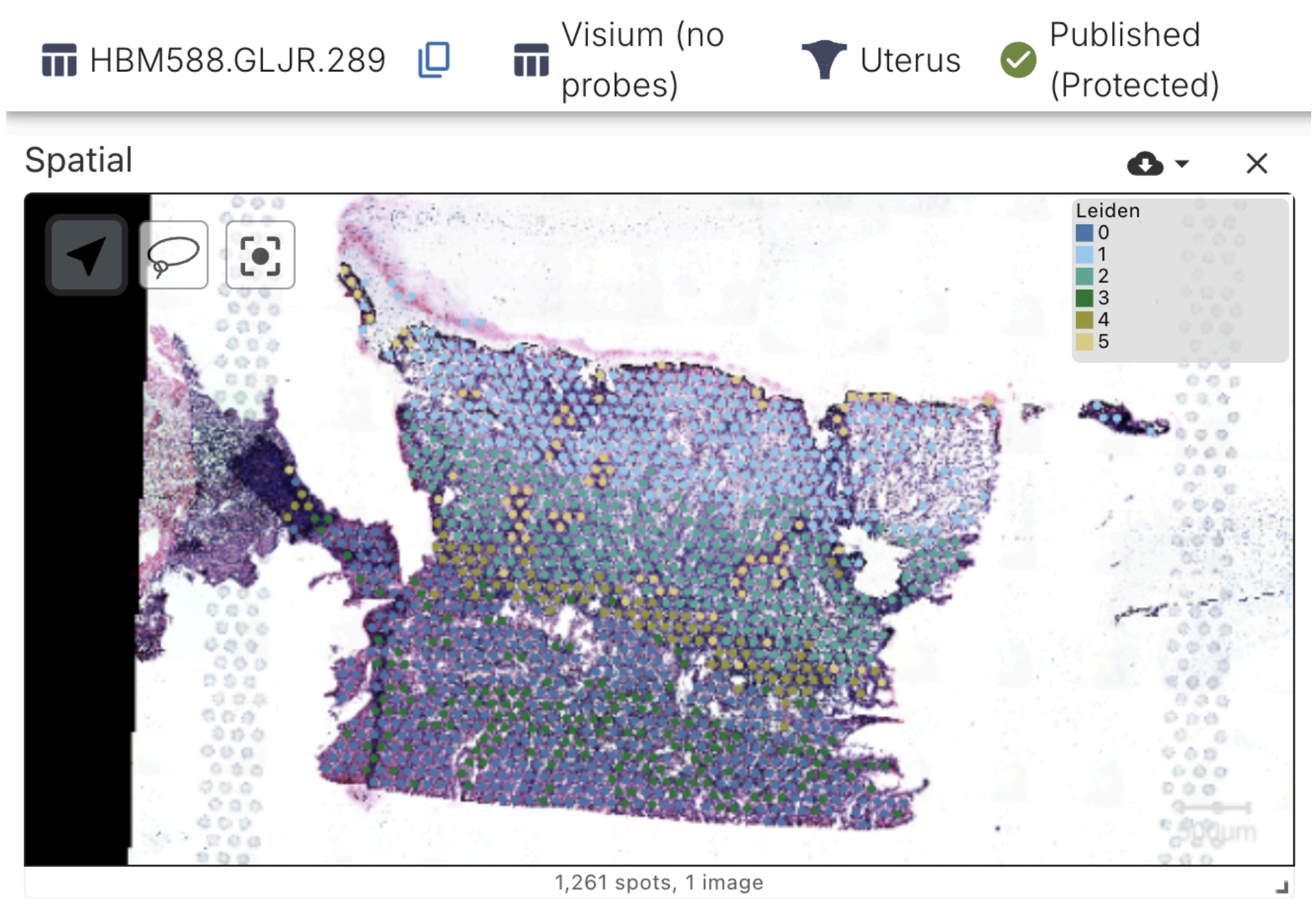

Within the HuBMAP Consortium, the ultimate responsibility for data quality lies with the data submitter, who has domain expertise in the technology and data type. Data submitters review processed datasets before publication, applying their own quality criteria beyond what is automated within the pipeline. Some variability in sample quality is an inherent possibility for a large multi-site consortium with multiple data generation centers each with their own unique organ-specific and technology-specific focus and expertise. If there are questions on other datasets, users are encouraged to contact the HuBMAP helpdesk and/or the original data submitter (contact details available on each dataset page).

2) In the main text (primarily Results section 1), we have clarified the data QA workflow to articulate who is responsible for data quality and the portal's role in transparently representing the entire processing history. We also encourage users to inspect datasets and apply their own quality criteria, emphasizing that visualizations are there for consumers to inspect the datasets directly in the portal before downloading or other use.

4. All published scRNA-seq or scATAC-seq datasets only have the Leiden cluster without annotation or have the annotation from the submitter. How are those unpublic data annotated? Based on submitter? If not and using other methods, please show some examples to ensure high quality annotation.

Changes made.

In the main text (primarily Results section 1), we have revised the language to ensure clarity on how cell types are identified and labeled.

Cell type annotations are systematically generated for sc/snRNA-seq datasets on supported tissues (currently heart, lung, and kidney) using Azimuth or Pan Human Azimuth

(https://azimuth.hubmapconsortium.org/), a reference-based cell type annotation tool. Currently, these are the only cell type annotations available on the portal. All cell type annotations methods are visible and searchable on the Datasets Search page (image below). Additional submitter or lab-based cell annotation methods generated outside of unified processing pipelines will also appear in this section once the data is publicly available.

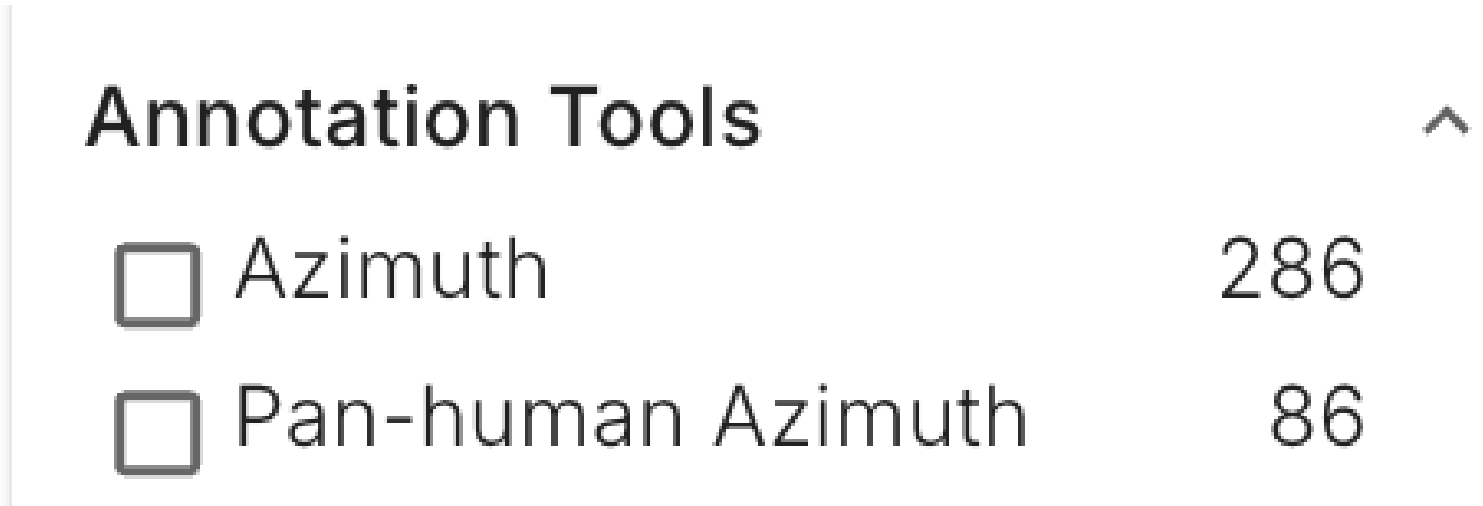

Leiden clustering is a purely computational, unsupervised step in the processing pipeline and does not depend on annotations provided by the data submitters. Because Azimuth is only applicable to a subset of datasets, some datasets will include only Leiden clustering, while others will include both Leiden clustering and Azimuth cell type annotations.

On dataset pages, cell type annotation information is visible under *Processed Data > Summary > Annotations*. The image below uses the example HBM822.JFXK.683 to show where annotation information appears on the dataset page and within the visualization. Any cell type annotation methods, when present, will appear in this section.

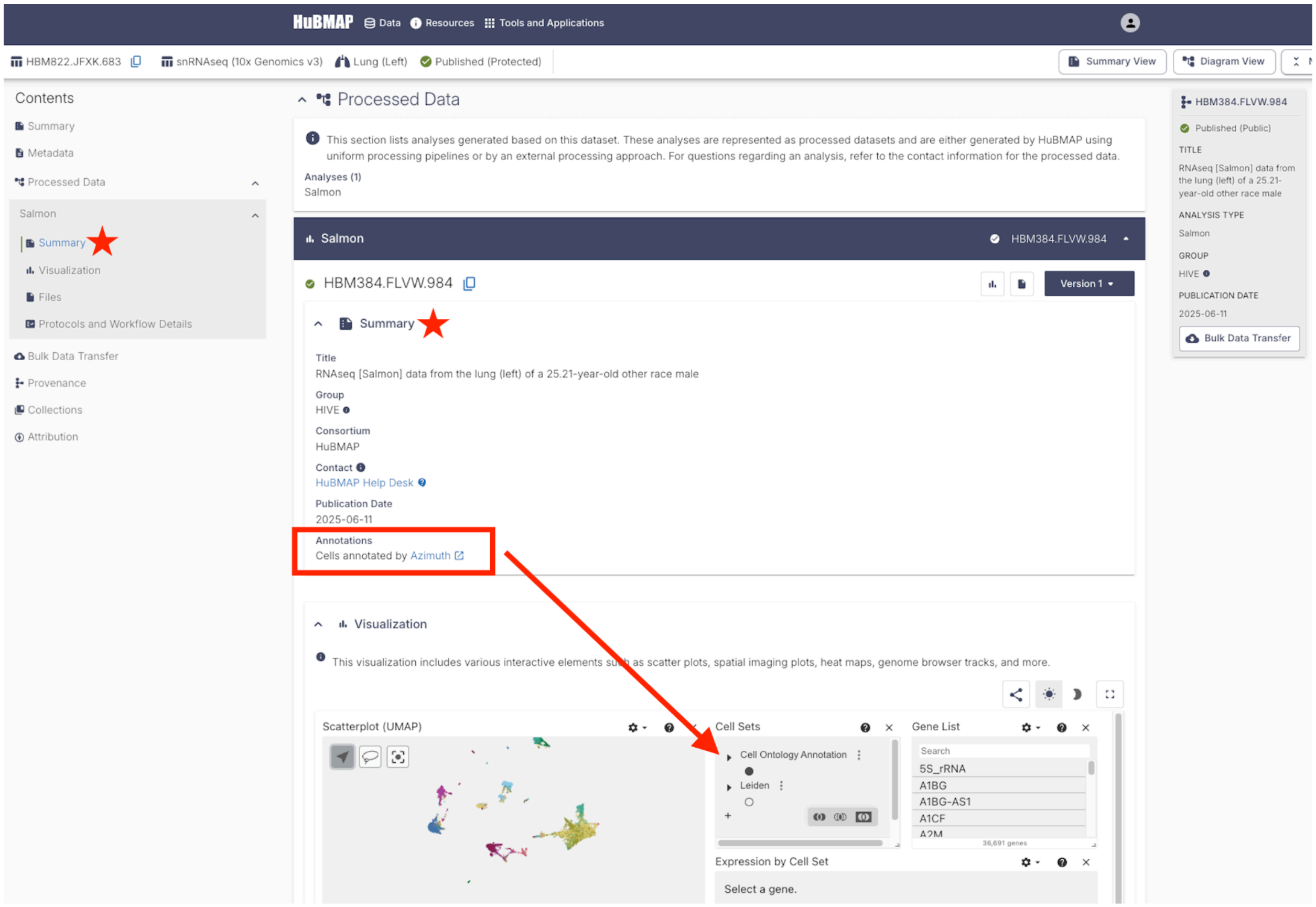

5. Some of the citations didn't match the text. For example, Azimuth was cited but the reference links to a paper that seems to have nothing to do with Azimuth. Please check all citations to ensure accuracy.

Changes made.

We reviewed all citations and updated the Azimuth reference to Hao et al., 2024, which describes the current version of Azimuth used in HuBMAP pipelines.

6. They state that researchers can use the Data Portal or request a resource from PSU to analyze the data. Please provide details, such as any approval needed, expected time length of data requested approved.

Changes made.

We revised the main text (Results, section 5) to include details on how to request resources from PSC to analyze the data.

7. The website is not very stable when visualizing large spatial datasets. If one dataset visualization is interrupted, other parallel datasets are also affected.

Changes made.

To improve performance when visualizing large datasets, we made several infrastructure updates: network improvements reduced data transfer times and visualization load times improved from 3.7 seconds to 0.5 seconds. The UI was also upgraded to React 19 for overall performance gains. HuBMAP infrastructure teams are actively investigating further solutions to reduce bandwidth consumption, including bot blocking and rate limiting. No changes were made to the main text as these are infrastructure updates rather than changes to the resource description.

Reviewer #2

Reviewer #2 (Remarks to the Author):

I co-reviewed this manuscript with one of the reviewers who provided the listed reports. This is part of the Nature Communications initiative to facilitate training in peer review and to provide appropriate recognition for Early Career Researchers who co-review manuscripts.

Reviewer #2 (Remarks on code availability):

They provide detailed and organized code. And they also provide R and jupyter notebook script example for users to practices.

No changes made.

Reviewer 2 remarks do not include any suggested revisions. We therefore did not make any changes in response to this comment.

Reviewer #3

Reviewer #3 (Remarks to the Author):

This manuscript describes the development and current state of the HuBMAP Data Portal as a repository for multi-modal spatial and single-cell data from healthy human tissues. The authors detail the infrastructure, including uniform processing pipelines, visualization, and cloud-based workspace environments. The scale of the engineering effort is impressive, hosting over 5000 datasets and providing a valuable centralization of complex spatial omics data. The manuscript is well-structured and documents a resource that will likely be important for the spatial biology community.

Overall, I find the resource description strong and the infrastructure robust, but there are significant issues regarding the framing of the data's representativeness and the clarity of its presentation that should be addressed before publication. My main concerns are as follows.

1. The manuscript emphasizes the utility of 'uniform processing pipelines' for harmonizing data across the portal. However, the distinction of processing between bulk, single-cell transcriptomics, and spatial proteomics is not made sufficiently clear, leading to potential confusion regarding the analysis provided. The text notes that cell type annotations were generated using Azimuth, but explicitly links this only to 'RNA-seq and Slide-seq' datasets. This implies that the vast majority of spatial datasets (e.g., CODEX, PhenoCycler and Visium) lack uniform cell type annotation. Tools like Azimuth are designed for transcriptomic and non-imaging data annotation. If the authors intended to imply that 'uniform processing' extends to spatial proteomics, they must clarify what specific methods were used, as transcriptomic tools are not directly applicable to imaging data. If a uniform strategy was indeed attempted for spatial data, there is a high risk of suboptimal annotation. Even between single-cell and bulk data, the “uniform pipeline” is different. For bulk data for example, cell-type deconvolution is a commonly used tool but it is certainly not needed for single-cell. Please explicitly state in the Methods or Results whether and what methods bulk, single-cell and spatial data undergo, respectively. Please qualify the term 'uniform processing' to avoid the misleading impression that spatial datasets possess the same level of annotation as the single-cell RNA datasets.

Changes made.

The main text was updated to clarify the following:

- Qualification of “uniform processing” terminology: Updated the Abstract and Introduction to clarify that uniform processing is data-type specific.
- Distinction of processing between bulk, single-cell, and spatial data types: Updated Results section 1 to note that processing steps vary by data type category, and added a new paragraph to the Methods (under Processing) providing an explicit breakdown of

pipeline steps for bulk, single-cell, and spatial data types, respectively.

- Clarification of Azimuth scope and annotation coverage: Updated Results Section 1 to clarify that Azimuth is a transcriptomic annotation tool applied only to sc/snRNA-seq, 10x Multiome, and Slide-seq datasets for supported tissues (heart, lung, and kidney). The new Methods paragraph explicitly states that spatial proteomics datasets undergo segmentation and unsupervised clustering via SPRM, and that uniform cell type annotation is not currently applied through the pipeline for these data types.

2. The labeling of data types in Figure 2h and Figure 3 is imprecise and potentially confusing for a technical audience. The authors use the label RNAseq to describe a category that appears to largely consist of bulk, single-cell and single-nucleus RNA sequencing data. Normally, RNA-seq implies bulk sequencing. Furthermore, the distinction between multimodal assays and their constituent parts is ambiguous in the bar plots. It is unclear whether the ATACseq bar includes the chromatin accessibility component of multimodal assays like SNARE-seq2 or 10x Multiome, or if these are counted separately. The figures should be relabeled to explicitly distinguish between bulk and single-cell modalities, and the caption should clarify how multimodal datasets are quantified to avoid perceived double-counting.

Changes made.

We revised the main text and figure captions of Figures 2 and 3 to address the concerns about label precision.

- RNAseq category clarification: The RNAseq label reflects how data types are organized on the HuBMAP Data Portal, which groups bulk, single-cell, and single-nucleus RNA sequencing data together as a single category. While we acknowledge that "RNA-seq" conventionally implies bulk sequencing in some contexts, HuBMAP uses the term broadly to refer to all assays that measure RNA abundance via sequencing. We clarified this in the main text (Results Section 1) and in the Figure 3 caption.
- Multimodal dataset counting: We added a clarifying note to both figure captions stating that multimodal assays are counted as distinct data types and are not disaggregated into their constituent assay components.

3. The manuscript describes the repository as a spatial reference atlas, but it lacks critical descriptive metadata regarding the nature of the spatial samples. For example, the text does not distinguish between Whole Slide Images (WSI), Biopsies, and Tissue Microarrays (TMAs). A 'spatial atlas' constructed primarily from TMAs (which lack anatomical gradients and macro-structure) has very different utility compared to one built from whole-tissue sections. Please clarify in the text and metadata figures (e.g., Figure 2) the proportion of spatial datasets that are TMAs vs. whole sections. Similarly, for each dataset, the authors should also clarify how many slices there are. Overall, more detailed statistics for spatial data is needed.

Changes made.

We have added clarifying text to Results Section 1 to address the reviewer's concerns about the nature of spatial samples in the portal.

- **WSI vs. TMA distinction:** HuBMAP spatial data primarily consists of imaged whole tissue sections and blocks representing contiguous anatomical regions, consistent with the program's goal of spatially resolved single-cell mapping of healthy human tissue. Portal samples are categorized as sections, blocks, organs, and suspensions, with tissue sections and blocks representing the majority of spatial data. TMAs comprise a minority of the current public HuBMAP data (13 public datasets out of 9,454). Additionally, the tissues within the TMAs contain spatial context and spatial visualizations. We have added a sentence to Results Section 1 clarifying HuBMAP's sample category framework (sections, blocks, organs, and suspensions) and the predominance of whole tissue data.
- **Slice count:** For datasets comprising multiple sections, the number of sections is available in dataset-level metadata under "number_of_sections," accessible on individual dataset pages in the portal (see example in image below of dataset HBM778.VHHR.349). A system-wide summary of slice counts across all spatial datasets is not currently available as a portal-level statistic.
- No changes made to Figure 2, as the changes to the main text address this concern by clarifying the predominance of whole tissue data on the portal.

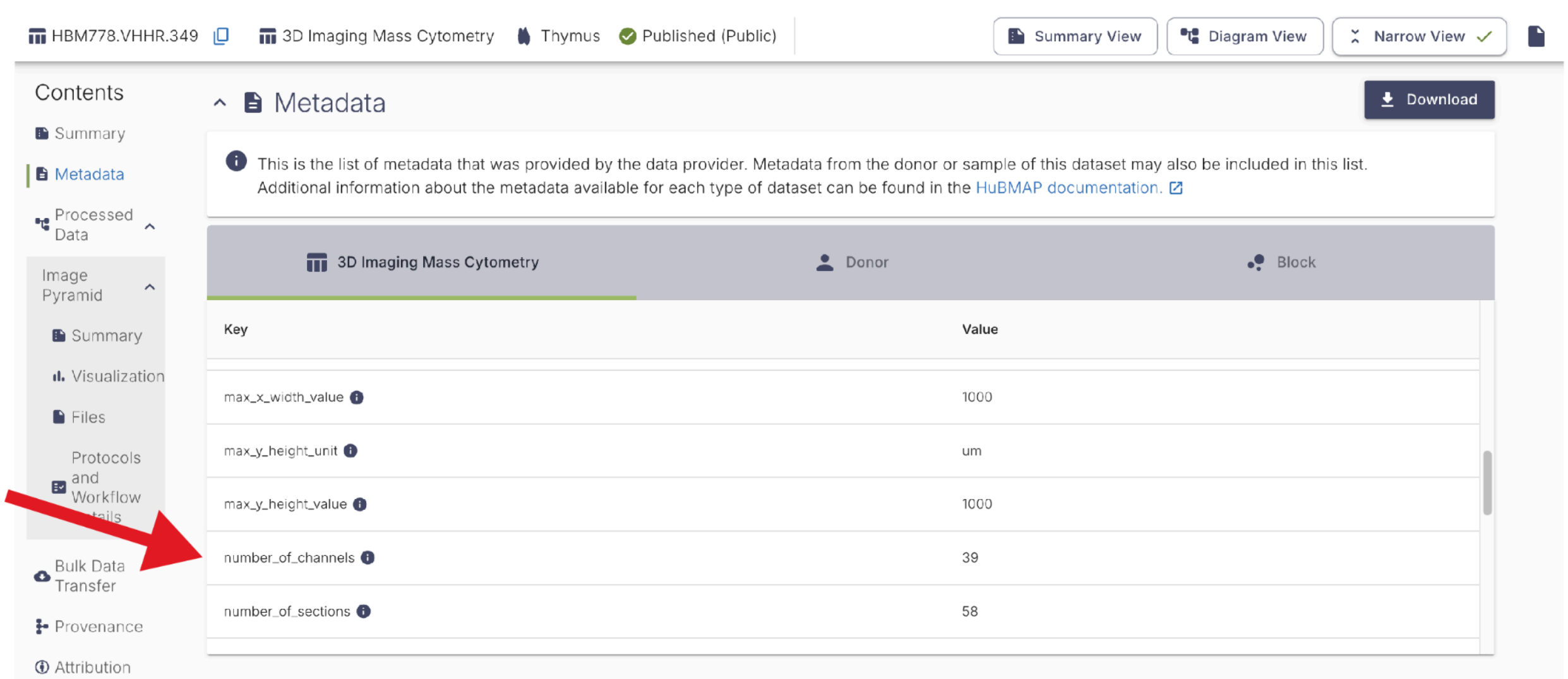

4. The manuscript lacks a sufficient discussion of the limitations imposed by the data skewness such as demographic (a big amount of samples are labeled as “unknown” and overwhelmingly, most samples are “white”). While the Discussion section touches on spatial sparsity, it glosses over the biological implications of the demographic imbalances. For a resource intended to

serve as a healthy reference for disease studies or a training set for computational models, the risks of using such a demographically narrow cohort as a control group are significant. I strongly suggest adding a limitation subsection that explicitly addresses these gaps and warns users about the potential artifacts arising from the lack of diversity and uneven organ sampling.

Changes made.

We revised the main text in two places to address the reviewer's concerns about demographic imbalances and data composition.

- Results Section 1: We added a sentence clarifying that donor composition reflects tissue availability and the research priorities of NIH-funded HuBMAP projects rather than a population-representative sampling strategy. Details on race and ethnicity metadata handling, including the "unknown" placeholder category, are provided in the Methods section and remain unchanged from the original submission.
- Discussion: We revised the opening of the limitations paragraph to explicitly acknowledge that the portal cohort does not fully reflect human demographic diversity and remains sparse in spatial coverage and data type representation across organs. We added a direct caution to users that these limitations should be considered when employing HuBMAP data as a healthy reference or training set, as findings may not generalize across all demographic groups or tissue contexts.

Overall, the HuBMAP Data Portal is a commendable technical achievement that advances the accessibility of spatial omics data. However, for the manuscript to serve as an accurate description of the resource, the authors must address the overstatements regarding the atlas's comprehensiveness and improve the precision of their data visualization labels. With these clarifications, the paper would be a valuable contribution to the field.

Reviewer #4

Reviewer #4 (Remarks to the Author):

In the manuscript tittled ' HuBMAP Data Portal: A Resource for Multi-Modal Spatial and Single-Cell Data of Healthy Human Tissues' the authors present their biological data portal/repository containing data generated in the HuBMAP (NIH, US) program. Data bases and data searches (incl HuBMAP) have been extensively evaluated by the NIH and this reviewer mainly evaluates the manuscript and the provided information, the novelty and impact and position within the landscape of life science data resources. This reviewer is not a member of the HuBMAP consortium and has not used the presented data portal prior to this evaluation task and only evaluated the examples provided by the authors.

Impact and gap: Major challenges in data driven life science are findability, standardization of data acquisition and (primary) analysis, quality control and availability of meta data on the origin of the used samples. There are several data repositories (GEO database, Array express) and data portals available including (public, non-profit) efforts that can search across multiple repositories like OmicsDI (EU). It is important that novel initiatives are not 'yet another data resource' but provide additional benefits and improvements to facilitate high quality life science research now and in the future. The collected data is impressive (methods, datasets) but is restricted to data generated within the HuBMAP consortium which has the advantage of standardized methods, sample description QC etc. The disadvantage of this restriction are the limited possibilities to apply HuBMAP data analysis and data visualization tools combining data from various resources (though implemented tools are opensource).

The manuscript is well written, but sometimes the detailed information is less relevant for readers not member of the HuBMAP consortium and there are several topics that would benefit from more information. Some of these are listed below. This manuscript/report could benefit from some use case examples.
Changes made in response to individual Reviewer 4 comments below. See those responses for details**.**

Use case examples are documented in Table 1. The table includes distinct use cases covering scientific goals, primary target users, portal tools, and impact across five use case categories derived from user studies. This table was expanded to include additional use cases that align with the manuscript, now up to 13.

1) HuBMAP data on the portal: provides 22 different data types of in total 5000+ samples.

- How can a user search for target genes in antibody and/or probe-based assays based on gene or protein panels? Could the authors describe how to get perform a gene-centric search finding datasets that contain information on specific genes or gene products.

No changes made.

Gene-centric search functionality, including searches by gene, protein, and Reactome pathway, is described in detail in Results Section 3 under "Biomarker and Cell Type Search." This functionality is compatible with both antibody-based and probe-based assays. For example, gene symbol can be used to search HuBMAP data and metadata,.

- Why do the authors include race in donor metadata as in biology human races do not exists? Ethnicity would be a more acceptable term.

No changes made.

The use of "race" in donor metadata follows NIH reporting standards, which require the collection of race and ethnicity as distinct categories for NIH-funded research. These are separate concepts in the NIH framework, where race and ethnicity are collected and reported independently. Using "ethnicity" in place of "race" would be inconsistent with these requirements. The use of "race" in this context does not imply a biological basis for race, but rather reflects administrative and regulatory reporting standards.

2) Data-types:

- It is difficult to find the rationality behind the choice for samples, number of samples and what methods applied to selection of tissue samples. Even though this is a large collection of samples; some tissues are overrepresented (780 right lung samples) where other tissues, like liver and brain, have <20 samples. This affects the completeness of the resource and the applications/ in silico experiments that will be biased by available data. Could the authors present an example analysis types of data mining/analysis that could be performed with the HuBMAP resource.

Changes made.

We revised the main text to address the reviewer's concerns about sample selection rationale and tissue representation.

- Sample selection rationale and tissue representation: The rationale for data composition on the portal, including uneven tissue representation, is addressed in revisions made in response to a related reviewer comment (R3 comment 4). Text was added to Results Section 1 and the Discussion clarifying that donor and tissue composition reflects the research priorities of NIH-funded HuBMAP projects and tissue availability, rather than a systematic population- or organ-representative sampling. Users are cautioned to consider these limitations when using HuBMAP data.

- Example analyses: The portal supports a range of data mining and analysis workflows through several integrated features. Publication pages (https://portal.hubmapconsortium.org/publications) provide examples of analyses performed on HuBMAP data, with interactive visualizations and links to associated datasets. Workspaces (https://portal.hubmapconsortium.org/workspaces) provide 15 pre-built Jupyter Notebook templates demonstrating analysis workflows across multiple data types. The Biomarker & Cell Type Search interface supports data-driven queries across transcriptomic, epigenomic, and proteomic data. These features are described in detail in Results Sections 3 and 5. The HuBMAP resource is well-suited for cross-organ cell type comparisons in organs with substantial representation, including placenta, lung, uterus, kidney, small intestine, and large intestine.

- Why are CODEX and Phenocycler data annotated differently?

Changes made.

We added clarifying text:

- We clarified in Results section 2 why they are separate data types on HuBMAP: "CODEX was recently rebranded to PhenoCycler, which is treated as a separate data type as it differs substantially in hardware and resulting data structure, imaging area, and biomarker capacity."
- Figure 5 was also updated to represent both CODEX and PhenoCycler visualizations on the portal.

3) Data Submission:

- Since only members of the HuBMAP consortium can upload data it is not that interesting for the reader/user not part to consortium to read the details about data submission. I link to the protocols would be sufficient. It would also be a great value for the reader/user to learn if some HuBMAP procedures are adapted from already used standards for data sharing.

Changes made.

We agree that submission details are primarily relevant to consortium members. To better serve non-consortium readers, we have condensed submission-specific detail in the main text and moved some content (e.g., donor metadata details) to the Methods. Non-consortium readers are now directed to https://docs.hubmapconsortium.org and https://protocols.io for full data submission documentation. The Data Submission section is retained in the methods to support reproducibility and transparency. We also added a data submitter use case to Table 1 to more clearly delineate the intended audiences for different portal sections, and added references to Fisher et al., (2026) and related portal efforts that document community standards development.

4) Data Ingestion:

- This step includes validation, reorganization, processing, quality assurance and quality control. The authors should also explain their procedures for implementing new standards in processing and quality control. In the Provenance paragraph the authors describe that data is versioned if data will be processed using new pipelines. It would be of great help for the reader to understand how this will be applied. Will all data be reprocessed at the same time to ensure the compatibility?

Changes made.

We expanded the Provenance section of the Methods to clarify that pipeline updates trigger selective rather than full dataset reprocessing; that pipeline versions are tracked and visible on individual dataset pages; and that users combining datasets processed at different times should check pipeline versions for compatibility.

5) HuBMAP data organization:

- Multi-omics data integration is extremely challenging and even integration of similar data like single cell or single nuclei transcriptomics data from different experiments is challenging. It is appreciated that HuBMAP is providing data integration and data analysis pipelines. The authors should provide an example on how their pipeline could and should be used and how the results should be interpret and benchmarked for accuracy.

Changes made.

We revised the opening of the Processing section in the Methods to clarify that HuBMAP's uniform processing pipelines are internal tools that produce harmonized, standardized outputs (AnnData/H5AD, OME-TIFF, SpatialData) compatible with community integration frameworks such as Scanpy and Seurat. For guided analysis examples, HuBMAP Workspaces provide 15 pre-built Jupyter notebook templates demonstrating workflows across multiple data types. Pipeline code is publicly available at https://github.com/hubmapconsortium for reference and transparency, however, pipelines are not intended as user-facing integration tools.

6) User-drive portal design and tool functionality

- It is clear from the efforts that HuBMAP aims to serve the life science community. It would be extremely valuable if the analysis HuBMAP data that results in peer reviewed scientific publications could be reproduced within HuBMAP and the used pipelines would be available. What scientific publications by HuBMAP consortia members involving data in the portal has been processed using the available tools and pipelines. A list us these publications and pipelines would be extremely valuable for the community to get familiar with the benefits and options of the data portal.

No changes made.

The portal's Publications section (https://portal.hubmapconsortium.org/publications) links peer-reviewed publications to their associated HuBMAP datasets, interactive Vitessce visualizations, and publication metadata. Datasets linked within these publication pages each contain detailed processing information. Closer integration of publication analyses with Workspace templates is a valuable direction for future development that we will consider as the portal evolves.

Reviewer #4 (Remarks on code availability):

I have looked a parts of the documentation and code, but it was too much to investigate everything in full detail.

It is a huge collection of scripts and code with sometimes strange names that are a bit difficult to navigate. A worked out example of creating and running a pipeline to answer a biological question would help the user to navigate through the options.

No changes made.
We appreciate the reviewer's feedback on the codebase. We clarify that the processing pipelines on GitHub are internal infrastructure used by the HIVE to process HuBMAP data – they are not intended as user-facing tools for running custom analyses. The volume and complexity of the codebase reflects the breadth of data types and processing steps required to operate the portal at scale.

For users interested in analyzing HuBMAP data and answer biological questions, the recommended entry point is HuBMAP Workspaces (https://portal.hubmapconsortium.org/workspaces), which provide 15 pre-built Jupyter notebook templates demonstrating analysis workflows across multiple data types. These templates are designed as guided, biological-question-driven examples that allow users to interact with HuBMAP data programmatically without requiring knowledge of the underlying pipeline infrastructure. Pipeline documentation is available at https://github.com/hubmapconsortium and https://docs.hubmapconsortium.org for users who want to understand the processing steps applied to the data.

Reviewer responses for NCOMMS-25-81343B: *HuBMAP Data Portal: a resource for multimodal spatial and single-cell data of healthy human tissues*

REVIEWER COMMENTS

Point-by-point responses to reviewer comments appear in red throughout.

Reviewer #1 (Remarks to the Author):

The authors have satisfactorily addressed my concerns. The revised manuscript is significantly improved and more clear.

Thank you. No changes made.

Reviewer #1 (Remarks on code availability):

Due to the huge amount of code in the portal, we were not able to carefully review all the code. But we asked specific questions related to code-generated pictures and segmentation which were satisfactorily addressed.

No changes made.

Reviewer #2 (Remarks to the Author):

I co-reviewed this manuscript with one of the reviewers who provided the listed reports. This is part of the Nature Communications initiative to facilitate training in peer review and to provide appropriate recognition for Early Career Researchers who co-review manuscripts.

No changes made.

Reviewer #3 (Remarks to the Author):

Overall, the authors answered my concerns nicely. However, I do have a new concern when reading the response for reviewer 1.

In their response, the authors state only that cell nuclei boundaries are 'segmented using the DAPI channel.' This describes the nuclear input to segmentation rather than the segmentation method itself. Because DAPI is a nuclear stain, segmentation from this channel alone yields nuclear masks rather than whole-cell boundaries; accurate whole-cell segmentation typically requires an additional membrane or cytoplasmic channel (e.g., E-cadherin) to delineate cell borders. Notably, modern segmentation tools such as Cellpose and Mesmer/DeepCell are explicitly multi-channel and do not rely on DAPI alone. If the pipeline performs only DAPI-based nuclear segmentation, this represents a meaningful limitation for a spatial reference atlas, as restricting boundaries to the nucleus systematically mis-assigns membrane- and cytoplasm-localized marker signal and may compromise per-cell quantification, particularly in densely packed tissues. Please specify the segmentation algorithm used, the input channels it relies on, and whether the outputs are nuclear-only or whole-cell segmentations. If uniform segmentation is genuinely DAPI/nuclear-only, this limitation and its downstream impact on per-cell feature extraction should be explicitly stated in the Methods.
Changes made.

Thank you to the reviewer for flagging this ambiguity. We have added text at the end of the Methods > HuBMAP Data Organization > Provenance section in the manuscript to clarify:

> *"Processing information for any given HuBMAP dataset can be found under the Protocols & Workflow Details section on a dataset page. For datasets with cell or nuclear segmentations, details are included under the Segmentation Channels & Quality section directly below the dataset visualization."*

The processing pipelines segment two distinct boundary types for some spatial proteomics datasets:

1) nuclear boundaries, segmented from the DAPI channel (sometimes appears under other labels, such as Hoechst, depending on the submitter's experiment)
2) cell membrane/whole-cell boundaries, segmented using markers that vary by experiment

The segmentation channels used for both boundary types are displayed under the **Segmentation Channels & Quality** section, which appears just below the data visualization on the dataset page. An example of this is shown in Image 1 below of a Cell DIVE dataset (HBM747.SPWK.779), which displays "Nucleus Segmentation Channel (DAPI_INIT)" and "Cell Segmentation Channel (PCAD)", indicated with red arrows in the image.

This section also links directly (Image 1, red box) to the **Protocols & Workflow Details** section that appears lower on the same dataset page. The Protocols & Workflow Details section (Image

2) includes a description of the assay-specific segmentation pipeline, methods used, selection criteria, and supporting citations.

Image 1

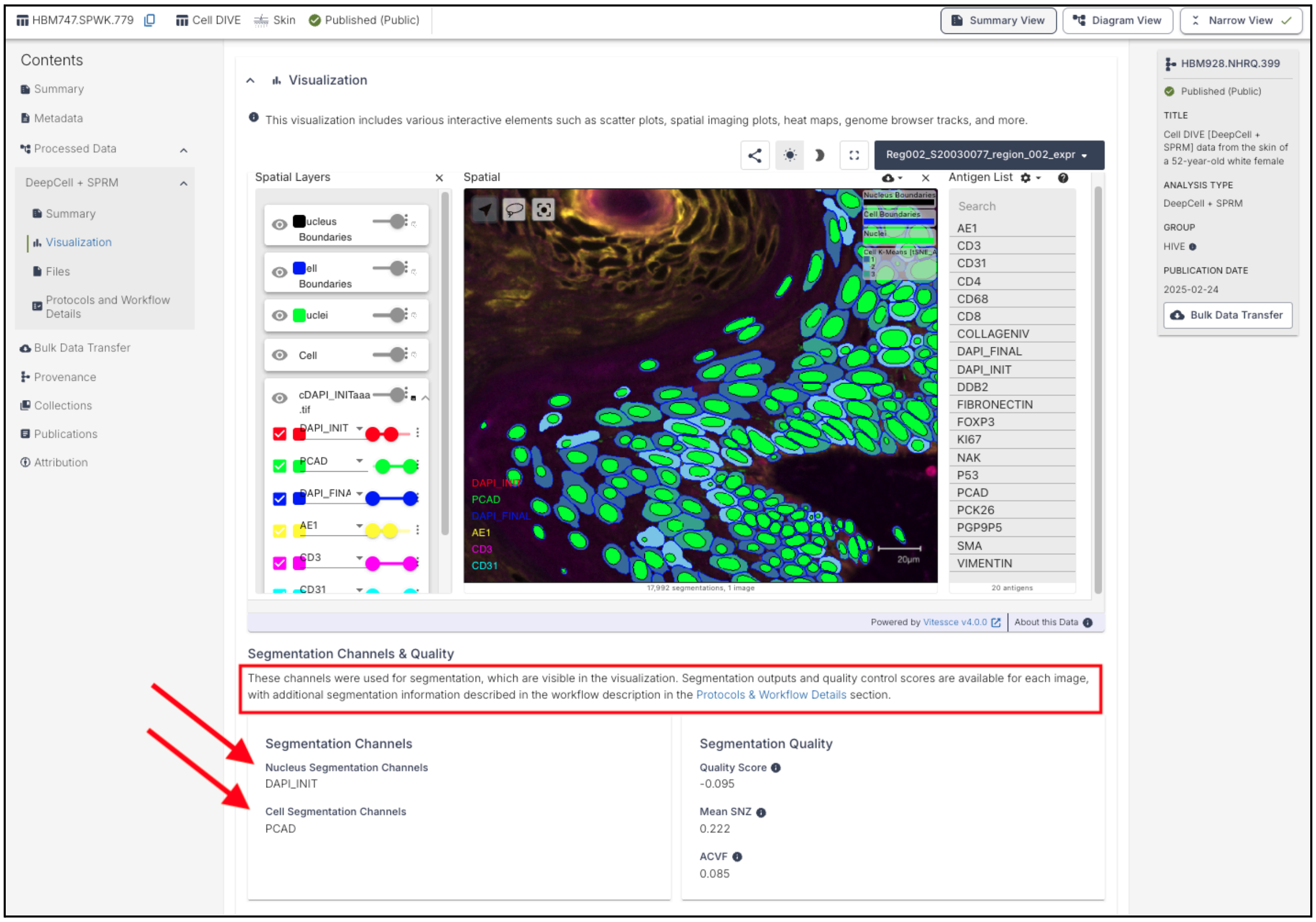

Image 2

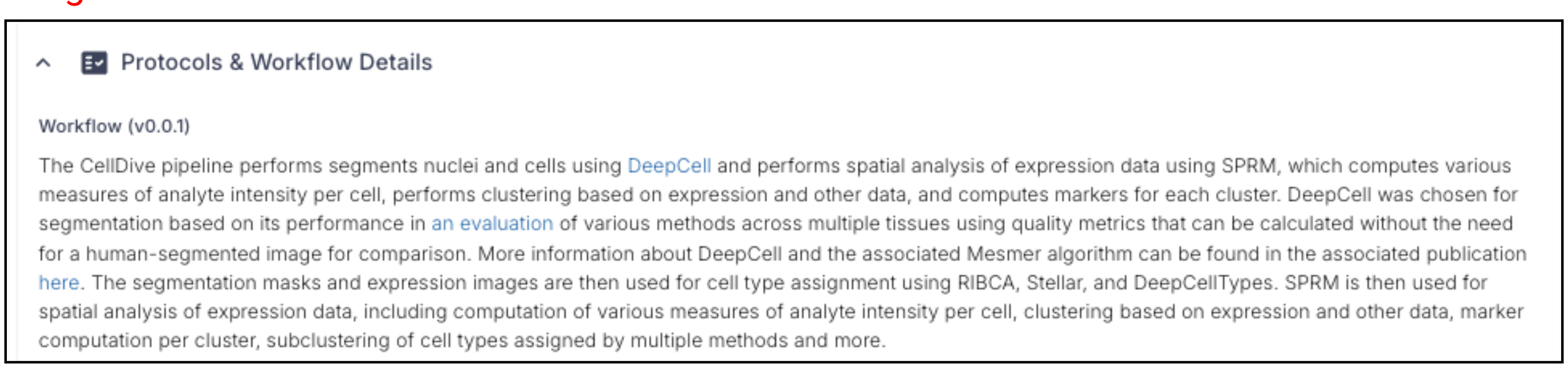

Protocols & Workflow Details

Workflow (v0.0.1)

The CellDive pipeline performs segments nuclei and cells using DeepCell and performs spatial analysis of expression data using SPRM, which computes various measures of analyte intensity per cell, performs clustering based on expression and other data, and computes markers for each cluster. DeepCell was chosen for segmentation based on its performance in an evaluation of various methods across multiple tissues using quality metrics that can be calculated without the need for a human-segmented image for comparison. More information about DeepCell and the associated Mesmer algorithm can be found in the associated publication here. The segmentation masks and expression images are then used for cell type assignment using RIBCA, Stellar, and DeepCellTypes. SPRM is then used for spatial analysis of expression data, including computation of various measures of analyte intensity per cell, clustering based on expression and other data, marker computation per cluster, subclustering of cell types assigned by multiple methods and more.

Reviewer #4 (Remarks to the Author):

In the revised version of the manuscript ‘HuBMAP Data Portal: A Resource for Multimodal Spatial and Single-Cell Data of Healthy Human Tissues’ the authors addressed all concerns raised by the reviewers. In my opinion the overall achievement, usefulness and impact on research are great and of great value. These types or large efforts need time to settle and mature, and the current version is not perfect but an has utility and is an excellent foundation to build on.

- I would like to apologize for requesting some information that was already available on the portal. The amount of information was a bit overwhelming, and I would like to thank the authors for their explanation and guidance.
- The use case table (table 1) is very helpful and inspiring on how to use the available data and tools in different life science research settings.
- Data submission paragraph, this now has all the information that a non-HubMAP user needs to understand the data submission process and assess the level and quality of the data.

With the development of AI and AI tools the landscape of life science is changing. This manuscript is a report on a development that started in 2018. The way researchers interact with data and the increasing use of AI agents has changed the data resource landscape and will chance the needs of users. It will be interesting to see the future development and directions of the HuBMAP project and data portal.

Thank you. No changes made.

Reviewer #4 (Remarks on code availability):

I have looked at the scripts in the repository, but as also indicated by the authors in their response this is not organized and optimized for sharing so I did not go into the details.

No changes made.
